# Cell-in-cell phenomenon associates with aggressive characteristics and cancer-related mortality in early oral tongue cancer

**DOI:** 10.1186/s12885-020-07342-x

**Published:** 2020-09-03

**Authors:** Alhadi Almangush, Antti A. Mäkitie, Jaana Hagström, Caj Haglund, Luiz Paulo Kowalski, Pentti Nieminen, Ricardo D. Coletta, Tuula Salo, Ilmo Leivo

**Affiliations:** 1 Department of Pathology, University of Helsinki, Haartmaninkatu 3 (P.O. Box 21), FIN-00014 Helsinki, Finland; 2 Research Program in Systems Oncology, Faculty of Medicine, University of Helsinki, Helsinki, Finland; 3 Department of Oral and Maxillofacial Diseases, University of Helsinki, Helsinki, Finland; 4 Institute of Biomedicine, Pathology, University of Turku, Turku, Finland; 5 Faculty of Dentistry, University of Misurata, Misurata, Libya; 6 Department of Otorhinolaryngology – Head and Neck Surgery, University of Helsinki and Helsinki University Hospital, Helsinki, Finland; 7 Division of Ear, Nose and Throat Diseases, Department of Clinical Sciences, Intervention and Technology, Karolinska Institutet and Karolinska University Hospital, Stockholm, Sweden; 8 Department of Oral Pathology and Radiology, University of Turku, Turku, Finland; 9 Research Programs Unit, Translational Cancer Medicine, University of Helsinki, Helsinki, Finland; 10 Department of Surgery, University of Helsinki and Helsinki University Hospital, Helsinki, Finland; 11 Department of Head and Neck Surgery and Otorhinolaryngology, A.C. Camargo Cancer Center, São Paulo, Brazil; 12 Department of Head and Neck Surgery, University of Sao Paulo Medical School, São Paulo, SP Brazil; 13 Medical Informatics and Data Analysis Research Group, University of Oulu, Oulu, Finland; 14 Department of Oral Diagnosis, School of Dentistry, University of Campinas, Piracicaba, São Paulo, Brazil; 15 Cancer and Translational Medicine Research Unit, Medical Research Center Oulu, University of Oulu and Oulu University Hospital, Oulu, Finland

**Keywords:** Cell-in-cell formation, Tongue neoplasms, Biomarkers, Mortality

## Abstract

**Background:**

Cell-in-cell structures (caused by cell cannibalistic activity) have been related to prognosis of many cancers. This is the first multi-institutional study to assess the prognostic impact of cell-in-cell structures in a large cohort of early oral tongue squamous cell carcinomas (OTSCC).

****Methods**:**

A total of 308 cases from five Finnish University Hospitals and from the A.C. Camargo Cancer Center, São Paulo, Brazil, were included in this study. Cell-in-cell structures were evaluated on surgical postoperative sections that stained with hematoxylin and eosin staining.

****Results**:**

We found that cell-in-cell structures associated with cancer-related mortality in univariable analysis with a hazard ratio (HR) of 2.99 (95%CI 1.52–5.88; *P* = 0.001). This association was confirmed in multivariable analysis (HR 2.22, 95%CI 1.12–4.44; *P* = 0.024). In addition, statistically significant associations were observed between the cell-in-cell structures and other adverse histopathologic characteristics including deep invasion (*P* <  0.001), high index of tumor budding (*P* = 0.007), worst pattern of invasion (*P* <  0.001), perineural invasion (*P* = 0.01), and stroma-rich pattern (*P* = 0.001).

**Conclusions:**

Our findings demonstrate a significant relationship between cell-in-cell formation and aggressive characteristics of early OTSCC. Cell-in-cell structures have a distinct impact as a novel prognostic indicator in early OTSCC and they can be easily assessed during routine pathology practice.

## Background

Oral cancer constitutes a major health problem with a global estimation of 354,864 new cases and 177,384 associated deaths in the year 2018 [1]. Oral tongue squamous cell carcinomas (OTSCC) is the most commonly reported carcinoma within the oral cavity and forms about one third of the diagnosed oral squamous cell carcinomas (OSCC) [2]. The incidence of OTSCC is increasing in many countries [3]. In addition, OTSCC associated with the highest cancer-related mortality compared with OSCC of the other oral subsites (floor of mouth, buccal mucosa, hard palate, gum, and retromolar trigone) [2]. Clinical behavior in many OTSCCs demonstrates an aggressive characteristic that associates with a moderate level of cancer-related mortality even in cases with early diagnosis [4]. In this context, many research efforts have been undertaken to introduce biomarkers that can help in early diagnosis by identifying oral tumors at an early stage when the lesion is small (≤ 4 cm in diameter), superficial and there is not yet metastasis [5, 6]. Clinically, such early stage tumors are usually referred to as cT1-T2N0 lesions. However, some of early-stage OTSCC have aggressive tumor behavior that requires multimodality treatment on a case-by-case basis. Unfortunately, it is challenging to identify those early OTSCC cases that require aggressive treatment if only conventional prognosticators (e.g. TNM stage, WHO grade or perineural invasion) are taken into consideration. Of note, research on prognostic biomarkers of cancer has not yet identified suitable candidates that could be considered in daily practice for the management of early OTSCC [7]. In addition, recent research has introduced new molecules as treatment targets for OTSCC [8, 9], although they are not yet clinically proven.

Invasion and metastasis are complex processes associating with cancer progression, and cancer tissues comprise dissimilar cell populations with variations in invasiveness and metastatic potential. Previous research has identified cellular and tissue prognostic markers related to characteristics of cancer behavior, such as apoptosis and tumor necrosis [7]. On the other hand, the clinical relevance of other mechanisms of cell death (for example cell cannibalism) have not been well-elucidated in early-stage OTSCC.

Cell-in-cell phenomenon/structure (also known as cell-in-cell formation, cancer cell cannibalism, in-cell invasion, or entosis) was described as a process of non-apoptotic cell death where one cancer cell surrounds another cancer cell followed by degradation of the internalized cell by lysosomal enzymes [10, 11]. This cell internalization process is different from phagocytosis as it is performed by a non-phagocytic cell, and the internalized cancer cell remains initially alive [12]. This phenomenon has been reported in various types of epithelial tumors and may promote tumor progression [13]. Histopathologically, a cell-in-cell structure has been defined as a larger cell enclosing a smaller cell within its cytoplasm. Cancer cell cannibalistic activity has been established as an important metabolic adaptation of cancers in an unfavorable microenvironment that lacking enough nutrition for cancer cells [13]. In addition, cannibalistic behavior has been shown to feed metastatic cells [13], where cannibalism was performed by metastatic and not by primary cells of melanoma [14]. It is noteworthy that the cell-in-cell structure has been well-known for decades among pathologists, but its clinical significance has remained neglected. Only recent research has emphasized the significance of the cell-in-cell phenomenon in the behavior of many cancers [15–17]. However, the clinical relevance of cell-in-cell has not been well-studied in many cancers of the head and neck region including early OTSCC.

The aim of the current study is to evaluate the prognostic significance of cell-in-cell structures in predicting cancer-related mortality in early OTSCC. We also aim to analyze the relationship between cell-in-cell structures and aggressive tumor features (e.g. worst pattern of invasion and perineural invasion) of early-stage OTSCC.

## Methods

### Patients

We included 308 cases treated for early OTSCC (cT1-2 N0) at the five university hospitals in Finland or at the A.C. Camargo Cancer Center, São Paulo, Brazil. Ethical approval was obtained from the ethics committees of each university hospital included and from the Finnish National Supervisory Authority for Welfare and Health (VALVIRA). For the Brazilian cases, a permission from the Brazilian Human Research Ethics Committee was obtained.

### Histopathologic evaluation of cell-in-cell structures

**Fig. 1:**
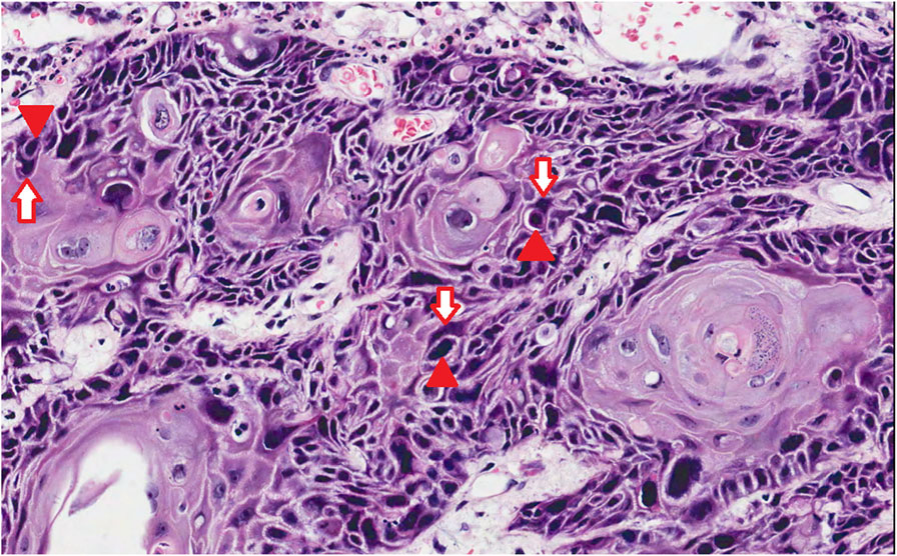
Cell-in-cell structures in early-stage oral tongue cancer. Winner cells (arrows) engulfing loser cells (arrowheads)

We used the surgical postoperative samples that were stained with hematoxylin and eosin (HE) for the assessment of cell-in-cell structures (Fig. 1). Cell-in-cell was defined as a structure consisting of a cancer cell contained inside another larger cancer cell with a crescent-shaped nucleus [15]. Such structures include also cancer cells with a morphological appearance of “bird’s-eye cells” and/or “signet-ring cells”. Low magnification (× 40 and × 100) was used to scan the whole sample. Structures including an ingested cell dislocating the nucleus of the other cell to the periphery of the structure were studied carefully with high magnification (× 200 and sometimes × 400).

Two observers (AA, IL) convened for a training session where examples of cell-in-cell were introduced by a researcher (AA), and the interpretation was guided by an experienced head and neck pathologist (IL). During the session, a discussion on the various shapes of cell-in-cell structures was conducted to standardize their recognition by the observers (AA, IL) and to assess randomly selected cases. The training session was followed by review sessions.

### Statistical analysis

We used IBM SPSS Statistics (version 25) for survival analysis and to calculate the prognostic significance of cell-in-cell. Univariable and multivariable survivals were estimated with hazard ratios (HR) and 95% confidence intervals (CI) using Cox regression. Kaplan-Meier survival curves were prepared to describe cancer-related mortality in association with the cell-in-cell phenomenon. The log-rank test was used to evaluate the statistical significance between the estimated curves. Cross-tabulation and chi-square test were used to analyze the association between cell-in-cell and other features of aggressive tumor behavior (e.g. tumor budding, depth of invasion, and tumor-stroma ratio) that we have evaluated in our previous research [18] (Table 1).

**Table 1:** Relationship between cell-in-cell structures and clinicopathologic features in early-stage oral tongue cancer

Variable	Total	Cell-in-cell		***P*** value of chi-square test
		None	One or more	
	***N*** = 308	*N* = 108 N (%)	*N* = 200 N (%)	
**Age**				0.397
≤ 60	128	41 (32.0)	87 (68.0)	
> 60	180	67 (37.2)	113 (62.8)	
**Gender**				> 0.999
Male	164	58 (35.4)	106 (64.6)	
Female	144	50 (34.7)	94 (65.3)	
**cTNM stage**				0.113
T1N0M0	123	50 (40.7)	73 (59.3)	
T2N0M0	185	58 (31.4)	127 (68.6)	
**Grade (WHO)**				**0.031**
Well differentiated	104	46 (44.2)	58 (55.8)	
Moderately differentiated	130	36 (27.7)	94 (72.3)	
Poorly differentiated	74	26 (35.1)	48 (64.9)	
**Tumor budding**				**0.007**
Low (< 5 buds)	212	85 (40.1)	127 (59.9)	
High (≥5 buds)	98	23 (24.0)	73 (76.0)	
**Depth of invasion**				**< 0.001**
Superficial (< 4 mm)	113	61 (54.0)	52 (46.0)	
Deep (≥4 mm)	195	47 (24.1)	148 (75.9)	
**Worst pattern of invasion**				**< 0.001**
Cohesive	77	41 (53.2)	36 (46.8)	
Invasive	231	67 (29.0)	164 (71.0)	
**Tumor-stroma ratio**				**0.001**
Low	220	90 (40.9)	130 (59.1)	
High	88	18 (20.5)	70 (79.5)	
**Perineural invasion**				**0.010**
Absent	267	101 (37.8)	166 (62.2)	
Present	41	7 (17.1)	34 (82.9)	

## Results

The main clinicopathologic features and their association with cell-in-cell phenomenon are summarized in Table 1. There were 200 tumors (64.9% of all cases) having cell-in-cell structures, while 108 (35.1%) had no cell-in-cell. Inter-observer agreement was good with Kappa value of 0.74.

The presence of cell-in-cell structures associated significantly with tumors with deep invasive growth (≥4 mm) (*P* <  0.001) and with tumors with a high frequency of tumor budding (*P* = 0.007). In addition, cell-in-cell structures were associated with worst pattern of invasion (*P* <  0.001), stroma-rich pattern (*P* = 0.001), WHO grade (*P* = 0.031) and perineural invasion (*P* = 0.01). We did not find any significant association between cell-in-cell structures and patient age (*P* = 0.397), gender (*P* >  0.999) or tumor size (*P* = 0.113).

**Fig. 2:**
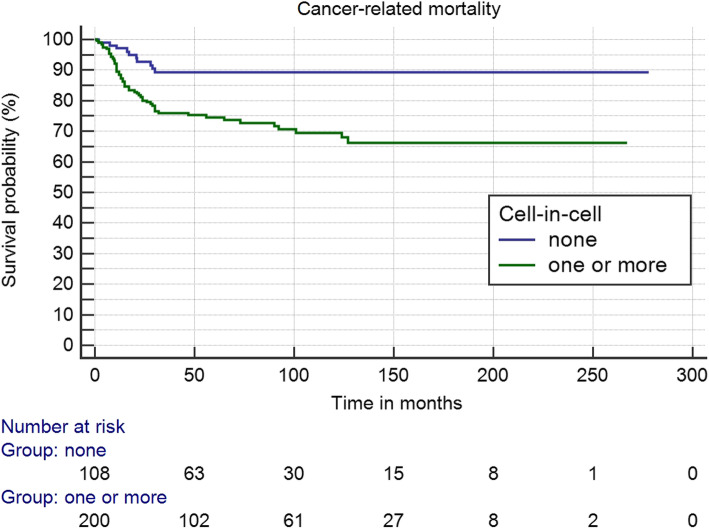
Kaplan-Meier analysis of cell-in-cell structures and patient survival. Cases with cell-in-cell structures associate significantly with a higher rate of cancer-related mortality (*P* < 0.001)

Univariable survival analysis showed a statistically significant prognostic value for cell-in-cell structures. Early OTSCC cases that have cell-in-cell structures displayed a higher rate of cancer-related mortality with a HR of 2.99 and 95% CI of 1.52 to 5.88 (*P* = 0.001). The prognostic value of cell-in-cell structures was confirmed in multivariable analysis (HR 2.22, 95% 1.12 to 4.44; *P* = 0.024) adjusted by age, stage, tumor grade, perineural invasion, worst pattern of invasion and depth of invasion. The multivariable Cox regression model revealed a significant effect of cell-in-cell structures on the survival independent from factors evaluated routinely (i.e. tumor grade, perineural invasion, pattern of invasion and depth of invasion) in pathology practice. In addition, the significance of cell-in-cell structures for prognostication of cancer-related mortality has been clearly shown by log rank test (*P* < 0.001) and Kaplan-Meier survival curves (Fig. 2).

## Discussion

The characteristics of invasiveness in each tumor type and stage vary from case to case. In early-stage OTSCC, many cases behave like advanced carcinoma and lead to cancer-related mortality. Moreover, in the same cancer tissue, cancer cells are also varying in their characteristics as some cells can be more aggressive than others. Identifying the aggressive cancer cells can help to recognize aggressive tumors. Cancer cells like unicellular organisms can engulf whole neighboring cells to scavenge for extracellular nutrients [13]. Such cannibalistic cancer cells have been assessed in this study and were found to have a significant association with aggressive behavior of early OTSCC.

**Fig. 3:**
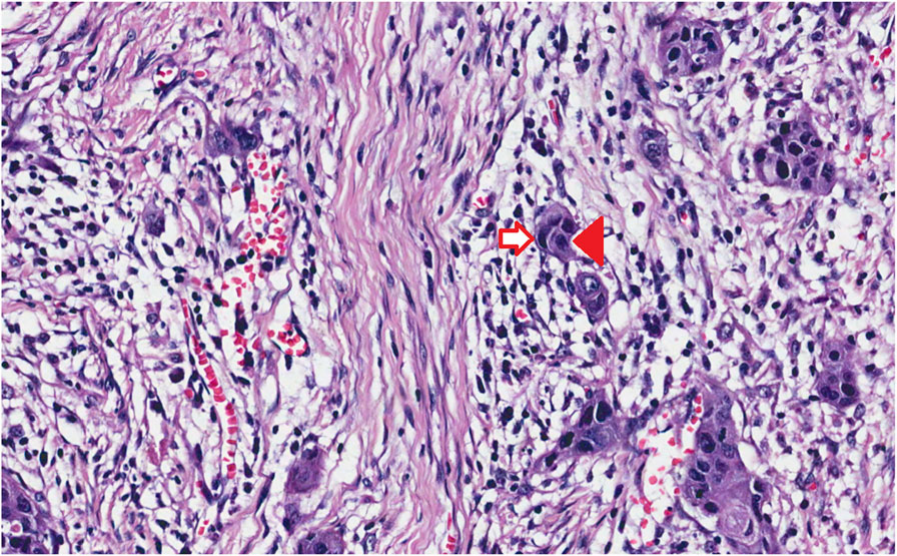
Association of cell-in-cell structures in early OTSCC with aggressive histopathologic features such as tumor budding (arrow). This is a deeply invasive tumor (> 4 mm) with a stroma-rich pattern

The clinical significance of cannibalistic cell-in-cell structures has been reported in many cancers as an adverse prognostic feature [16, 19, 20]. Cellular cannibalism (i.e. cell-in-cell structures) has been reported commonly in various cancers but not in normal tissues [16]. In addition, we found a significant association between cell-in-cell structures of early OTSCC and other aggressive histopathologic features that are tumor-related (e.g. tumor budding and depth of invasion) or stroma-related (e.g. tumor-stroma ratio) (Fig. 3). Moreover, metastatic cancers have been reported with a higher occurrence of cell-in-cell structures than non-metastatic cancers [21]. These facts propose that cellular cannibalism is a possible hallmark of an aggressive cancer. In a cohort of head and neck squamous cell carcinomas, Schenker et al. [22] found that formation of cell-in-cell structures had a superior prognostic value compared to apoptosis or senescence. Furthermore, Mackay and colleagues [19] reported that cell-in-cell formation was an independent prognostic marker in lung adenocarcinomas and had an association with the occurrence of mutant p53 and genomic instability in these tumors. In pancreatic ductal adenocarcinoma, Hayashi et al. [15] found that cell-in-cell structures predict prognosis, and associate with poorly-differentiated tumors, TP53 mutations, KRAS amplification and MYC amplification.

The mechanism underlying cancer cell cannibalism is somehow similar to phagocytosis as both processes can be against the apoptotic cells; however, cannibalistic cancer cell engulfs live cancer cells as well [13]. This behavior of the cannibalistic cancer cells and reasons that induce a cancer cell to invade its neighboring cancer cell has been an area of active investigation for many years. Similar to other cells, cancer cells need nutrient scavenging from their environment especially since tumor vasculature is deficient in many tumors [23]. In addition, cancer cells are known to compete for nutrients. During the formation of cell-in-cell structures, the engulfing cell (i.e. winner) cannibalizes the engulfed cell (i.e. loser) as an expression of competition between hungry cancer cells [24]. Moreover, Hamann et al. [25] have reported that the process of ingesting a neighboring cancer cell is initiated by glucose starvation, allowing for the proliferation of the winner cell. Thus, in low-nutrient environments, cannibalism was seen as a mechanism to support the proliferation of cancer cells [23]. Furthermore, Lugini et al. [14] found that cannibalistic activity increased cell survival of metastatic melanoma. Thus, it was speculated that cannibalism is a method for feeding metastatic cancers [26].

Multivariable analysis of the current cohort showed that cell-in-cell is an independent prognostic marker when adjusted for other factors including depth of invasion, pattern of invasion, tumor grade and perineural invasion. Interestingly, the above mentioned four parameters are reported often in pathology reports and, in a recent study on early-stage oral cancer [27], they were recognized as important prognosticators. This indicates that the cell-in-cell structure is a histopathologic characteristic providing prognostic information complementary to conventional prognostic features. Furthermore, studies in other cancers support our findings on the cell-in-cell structure as an indicator of aggressive behavior in OTSCC [15, 16, 28–31].

## Conclusions

Cell-in-cell structures can be used to identify a subgroup of patients with an aggressive early-stage OTSCC with a high rate of cancer-related mortality. The assessment of cell-in-cell structures can be conducted using HE-stained sections and they can be used as a new tool to determine the aggressiveness of early OTSCC. As the cannibalistic cell-in-cell structures associate with aggressive behavior of early OTSCC, targeting such cannibalistic activity might even form a platform for anti-cancer therapies. Future studies need to validate the findings of our current report, preferably in prospective cohorts. After validation, inclusion of the assessment of cell-in-cell structures in routine pathology reports should be considered. Furthermore, the molecular mechanisms underlying cell-in-cell structures in OTSCC should be addressed.

## Data Availability

The datasets used in this study is available from the corresponding author upon a reasonable request.

## Abbreviations

CI: Confidence interval
HR: Hazard ratio
HE: Hematoxylin and eosin
WHO: World Health Organization
OTSCC: Oral tongue squamous cell carcinoma
